# Insights into novel diagnostic assay development, antimicrobial resistance, and pathogenicity in *Proteus mirabilis* through pan-genome analysis

**DOI:** 10.1128/aem.01898-25

**Published:** 2026-02-24

**Authors:** Zhiqiu Yin, Xiaman Chen, Juncong Xiao, Xiaoyan Tian, Zhuolin Li, Mujie Zhang, Baixin Jing, Dongsheng Li, Xiaoyan Deng, Liang Peng

**Affiliations:** 1 Department of Clinical Laboratory, Key Laboratory of Biological Targeting Diagnosis, Therapy and Rehabilitation of Guangdong Higher Education Institutes, The Fifth Affiliated Hospital, Guangzhou Medical University 656261, Guangzhou, Guangdong, China; 2 KingMed School of Laboratory Medicine, Guangzhou Medical University 26468, Guangzhou, Guangdong, China; 3 Engineering Technology Research Center of Intelligent Diagnosis for Infectious Diseases in Guangdong Province, Guangzhou, China; 4 Guangzhou Key Laboratory for Clinical Rapid Diagnosis and Early Warning of Infectious Diseases, Guangzhou, China; University of Georgia Center for Food Safety, Griffin, Georgia, USA

**Keywords:** *Proteus mirabilis*, pan-genome analysis, species-specific core gene, TaqMan-based real-time PCR, antimicrobial resistance, virulence

## Abstract

*Proteus mirabilis*, a significant pathogen associated with human urinary tract infections (UTIs), demonstrates escalating multidrug resistance (MDR) that complicates clinical management. Accurate identification and in-depth genomic analysis are essential for monitoring and controlling this pathogen. This study aimed to identify the species-specific gene repertoire, antimicrobial resistance (AMR), and virulence genetic profiles through pan-genome analysis to develop novel detection methods and better understand emerging public health threats. The genus *Proteus* exhibits an open pan-genome, with *P. mirabilis* harboring a distinct species-specific gene repertoire. Two species-specific core genes, *PMI3020* and *PMI3598*, were identified as molecular targets. We developed conventional PCR and TaqMan probe-based real-time PCR assays, which demonstrated high specificity when tested against *P. mirabilis* and non-*P*. *mirabilis* isolates. The TaqMan probe-based real-time PCR demonstrated a sensitivity of 3.43 × 10² CFU/mL using serial dilutions of *P. mirabilis* DNA. Comparative genomic analysis revealed significant differences in AMR and pathogenicity-related gene repertoires between *P. mirabilis* and other *Proteus* spp. The higher prevalence of AMR phenotypes in *P. mirabilis* correlated with its greater abundance of AMR genes. Emerging AMR genes acquired through horizontal gene transfer (HGT) have increased MDR risks, particularly to carbapenems and cephalosporins. Additionally, *P. mirabilis* genomes contain more virulence genes mainly related to adherence and iron acquisition. Our findings establish pan-genome analysis as an effective tool for identifying specific genetic markers to detect pathogens accurately and provide a comprehensive genomic framework illuminating AMR dynamics and virulence in *P. mirabilis*, thereby providing a valuable foundation for future public health risk assessments.

**IMPORTANCE:**

*P. mirabilis* is a major uropathogen with increasing AMR prevalence. The dissemination of AMR genes across healthcare and community settings poses critical challenges to infection control. This study conducted pan-genome analysis of *Proteus* to identify *P. mirabilis*-specific gene repertoire, of which species-specific core genes were used as molecular targets to develop highly sensitive PCR assays for accurate detection of this pathogen. Compared with other *Proteus* spp., *P. mirabilis* possesses a greater abundance of AMR genes, resulting in a higher prevalence of AMR phenotypes, including significant resistance to carbapenems and cephalosporins. This study establishes pan-genome analysis as an effective strategy for mining species-specific genetic markers, enabling the development of novel PCR assays for accurate pathogen detection. The comprehensive genomic framework enhances understanding of AMR dynamics and virulence mechanisms essential for public health risk assessment.

## INTRODUCTION

*Proteus mirabilis* is a Gram-negative, facultatively anaerobic, rod-shaped bacterium belonging to the genus *Proteus* (1, 2). As a well-established uropathogen, *P. mirabilis* is frequently associated with urinary tract infections (UTIs), which typically progress to severe complications such as urinary tract obstruction, urolithiasis (bladder or kidney stones), catheter blockage, bacteremia, and life-threatening sepsis (3, 4). The emergence of multidrug resistance (MDR), defined as acquired non-susceptibility to at least one agent in three or more antimicrobial categories (5), in *P. mirabilis* has intensified, largely due to numerous clinically relevant antimicrobial resistance (AMR) genes. These genetic determinants confer both intrinsic resistance and acquired resistance mechanisms, making *P. mirabilis* a particularly challenging pathogen to manage, especially in the context of the global antimicrobial resistance crisis. Recent large-scale genomic studies have deepened our understanding of its diversity and clinical impact. Potter et al. revealed extensive subspecies divergence and lineage-specific accessory genes encoding virulence factors (6), while Lian et al. documented pervasive carbapenem/quinolone resistance and conserved urease clusters across global isolates (7). These advances underscore the critical need to investigate strain-specific adaptations driving pathogenesis and treatment failure.

Current identification methods for *P. mirabilis* remain limited, with conventional culture methods, selective media, biochemical tests, serological tests, 16S rRNA sequencing, and MALDI-TOF MS serving as the primary diagnostic tools (8, 9). However, traditional culture-based methods suffer from low sensitivity. Nowadays, molecular-based methods for pathogen identification have been developed, including conventional polymerase chain reaction (PCR), real-time PCR, loop-mediated isothermal amplification (LAMP), and microarray (10, 11). Among these, TaqMan real-time PCR is preferred for its rapidity, ease of use, and high sensitivity/specificity, allowing real-time monitoring via fluorescence (12, 13). The design of oligonucleotide probes and primers constitutes a critical aspect of TaqMan real-time PCR assays. While most real-time PCR methods target the 16S rRNA gene and housekeeping genes, the limited polymorphism of these sequences often precludes species-specific identification. The rapid expansion of genomic data has facilitated the widespread application of pan-genome and comparative genomic analyses for investigating genetic variation, gene function enrichment, and evolutionary dynamics (14, 15). These approaches have proven valuable for identifying novel biomarker genes with improved taxonomic resolution for pathogen detection (16, 17).

In this study, we performed whole-genome, phylogenetic, and pan-genome analyses on diverse *Proteus* species, focusing on the genetic relatedness, phylogenetic position, and species-specific genetic markers of *P. mirabilis*. Through comparative genomic analysis of the *Proteus* pan-genome, we identified novel *P. mirabilis*-specific core genes as molecular targets for developing accurate and sensitive TaqMan real-time PCR assays. Furthermore, we investigated the genetic basis of key characteristics within the pan-genome, including genotypic and phenotypic profiles related to AMR and virulence, to better understand the mechanisms underlying the emerging threats to public health and food safety posed by this species.

## RESULTS AND DISCUSSION

### Selection of high-quality representative genomes and evaluation of genetic relatedness

In this study, we collected all available genomes of the genus *Proteus* as defined by the NCBI RefSeq database (accessed on 14 November 2022), totaling 679 genomes (Table S1). We then applied stringent quality filters to define a high-quality genome, retaining only those with a contig number ≤ 200, completeness ≥ 99%, and contamination ≤ 10%. Recognizing that redundant strains with close genetic relationships could introduce ascertainment bias in pan-genome analysis (18), we selected representative genomes and excluded redundant ones (average nucleotide identity (ANI) > 99.8%), resulting in a high-quality representative genome data set (*n* = 259; contig number = 36.1 ± 38.7; completeness = 99.5% ± 0.1%; contamination = 0.1% ± 0.6%) of the genus *Proteus* (Table S1).

**Fig 1:**
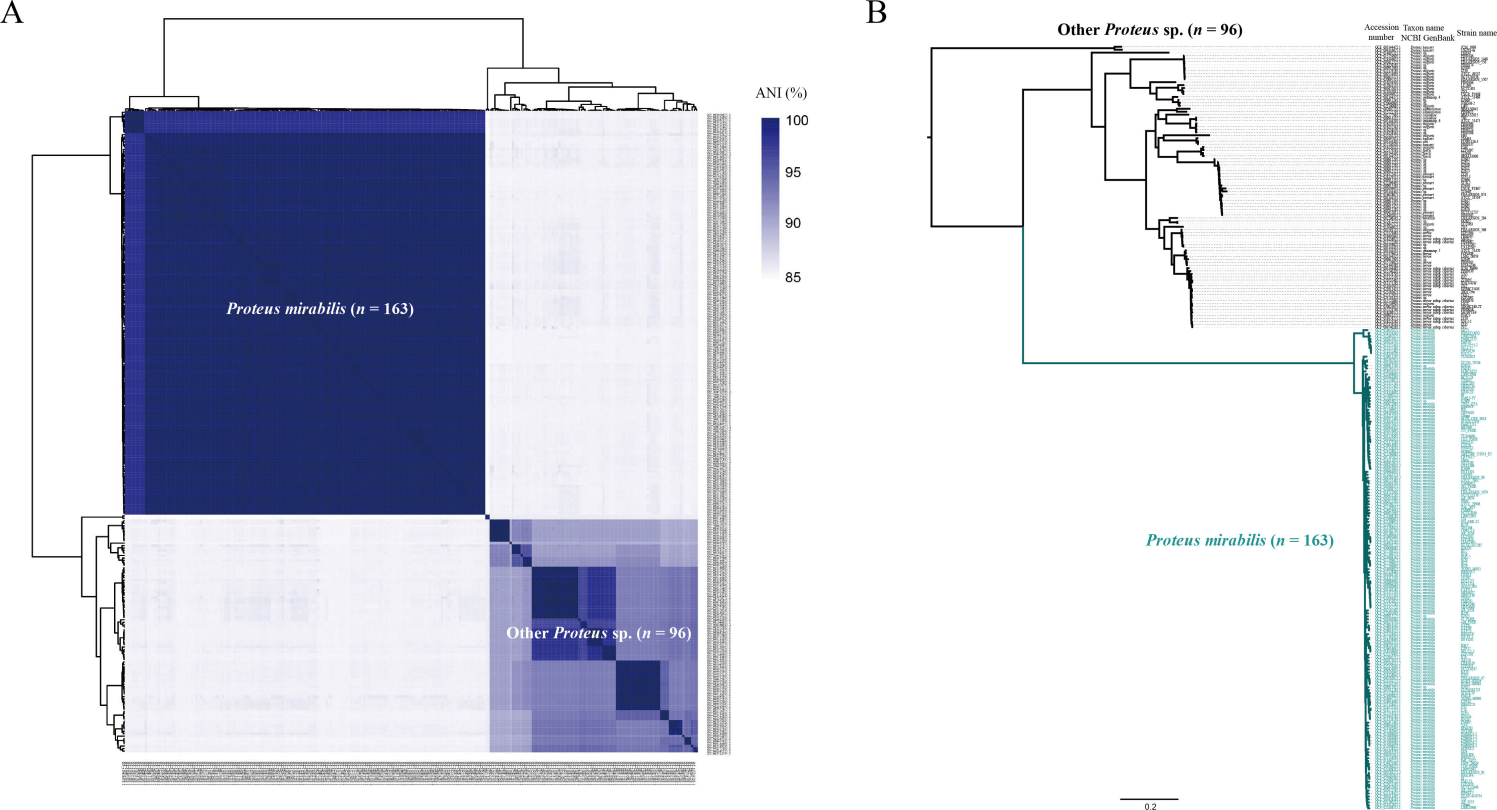
Genetic relatedness of *Proteus mirabilis* within the genus *Proteus*. (**A**) Pairwise average nucleotide identities (ANI) among the 259 *Proteus* spp. genomes, showing hierarchical clustering relationships. ANI values are represented by heatmaps, where similarity values are represented by the color key histograms on the right panels. (**B**) Core genome phylogeny. The maximum likelihood (ML) tree was constructed using single-nucleotide polymorphisms (SNPs) across 1,954 single-copy core gene families shared among 259 *Proteus* spp. genomes.

The ANI was used to measure the genetic relatedness between these genomes. As shown in Fig. 1A, the majority of these genomes (*n* = 163), including the reference genome HI4320, formed a closely related cluster with high ANI values (99.0% ± 0.7%), identified as *P. mirabilis*. The remaining 96 genomes were positioned outside this cluster. The average ANI values between *P. mirabilis* and other *Proteus* spp. were 85.3% ± 0.2%, which is below the 95% threshold typically used for species delineation (19). We performed a phylogenetic analysis based on single nucleotide polymorphisms (SNPs) across 1,954 single-copy core gene families shared by all 259 *Proteus* spp. genomes. In the core genome tree (Fig. 1B), all *P. mirabilis* strains formed a distinct monophyletic clade with substantial branch length, deeply nested within the genus *Proteus*. This clade diverged early in the phylogeny, showing a clear genetic distinction between *P. mirabilis* and other *Proteus* spp.

### Pan-genome architecture of the genus *Proteus*

**Fig 2:**
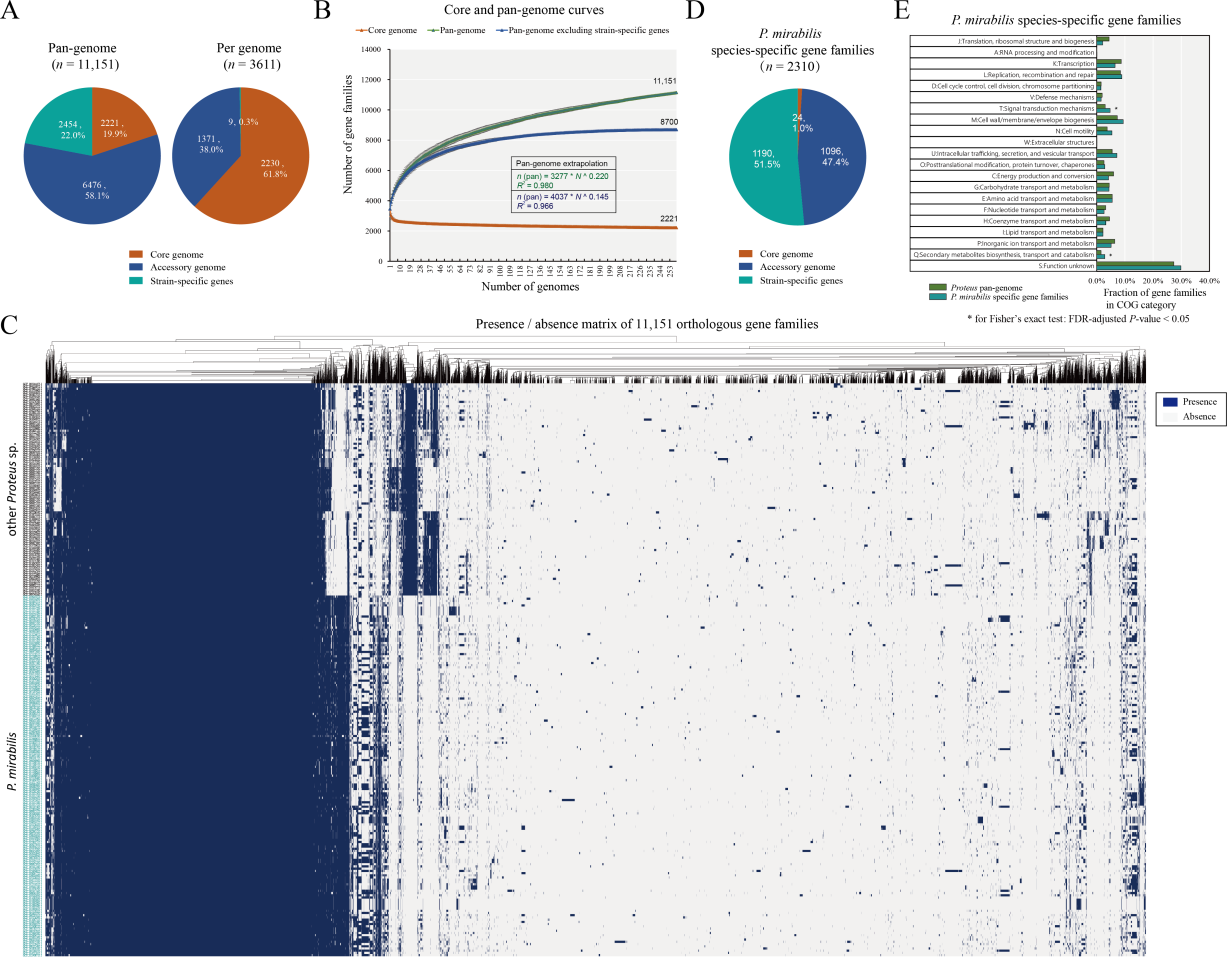
Pan-genome analysis of the genus *Proteus*. (**A**) Proportional distribution of pan-genome components. Pie charts depict percentage compositions of core, accessory, and strain-specific gene families in both the pan-genome and individual genomes. (**B**) Accumulation curves for core genome, pan-genome, and pan-genome excluding strain-specific genes. Core gene families trended to decrease with the addition of genomes, whereas the pan-gene families tended to increase. The inferred mathematical functions describing the pan-genome curves are shown in the graph. (**C**) Gene presence/absence matrix of the *Proteus* pan-genome. The left side represents the phylogenetic tree based on core genome. The right side represents the matrix where the genes were either present or absent. The presence of genes is represented by blue blocks, whereas their absence is denoted by gray blocks. (**D**) Composition of pan-genome components in *P. mirabilis* species-specific gene families. Pie charts depict proportions of core, accessory, and strain-specific gene families within these gene families. (**E**) Functional categorization of *P. mirabilis* species-specific gene families. Enrichment was assessed by Fisher’s exact test with Benjamini-Hochberg FDR correction; * for FDR-adjusted *P*-value < 0.05.

Pan-genome analysis represents a powerful method for obtaining the abundant genetic information of a bacterial genus/species (20). Here, a total of 11,151 homologous gene families were identified from the 259 *Proteus* spp. genomes, which constitute the *Proteus* pan-genome (Fig. 2A). Of these, 2,221 (19.9%) are present across all genomes, representing the core genome; 6,476 (58.1%) form the largest group and represent the accessory genome, being present in at least one genome; the remaining 2,454 (22.0%) are only present in one genome, representing the strain-specific gene content (Fig. 2A). On average, individual genomes contained 2,230.4 ± 4.7 core genes, 1,371.4 ± 182.6 accessory genes, and 9.5 ± 14.0 strain-specific genes. It is evident that the accessory gene families of the *Proteus* pan-genome are abundant, contributing to the genetic diversity of this genus. The accumulation curve for each additional genome was fitted to the Heaps’ power law function (*n* = $\kappa N^{\gamma }$) to ascertain whether the *Proteus* pan-genome is open (*γ* ≥ 0) or closed (*γ* < 0). An open pan-genome indicates that a genus or species has a high capacity to exchange genetic elements, whereas a closed pan-genome indicates a limited capacity to acquire foreign genes (21). The inferred growth exponent (*γ*) value of 0.2220 indicates that the *Proteus* pan-genome is open (Fig. 2B), suggesting that this genus has a gene pool that allows for the continuous acquisition of exogenous genetic elements. This open architecture is in alignment with recent findings in *P. mirabilis*, where lineage-specific accessory genes (e.g., secretion systems, prophages) have been shown to drive functional diversification across subspecies (6, 7). Furthermore, when strain-specific genes were excluded, a plateau emerged in the pan-genome accumulation curve. The core genome curve demonstrates that the size of the core genome decreases with the addition of genomes (Fig. 2B).

### *P. mirabilis* species-specific gene repertoire

According to the absence/presence profile of orthologous gene families, we identified the *P. mirabilis* species-specific gene repertoire, which is defined as gene families present in *P. mirabilis* genomes but absent in other *Proteus* spp. genomes (Fig. 2C). This repertoire was categorized into three groups: (*P. mirabilis* species-specific) core (genes shared among all 163 genomes), accessory (genes shared among more than 1 and less than 163 genomes), and strain-specific (genes that existed in only 1 genome) gene families. The analysis revealed 2,310 gene families (Fig. 2D), comprising 24 (1.0%) core, 1,096 (47.4%) accessory, and 1,190 strain-specific gene families. This finding indicates that most of the *P. mirabilis* species-specific gene families are not core but are variably shared, showing tremendous strain-level diversity. These gene families are significantly enriched in the cluster of orthologous group (COG)-T (*n* = 51; 4.7%; Signal transduction mechanisms) and -Q (*n* = 31; 2.9%; secondary metabolites biosynthesis, transport, and catabolism) [Fisher’s exact test: False Discovery Rate (FDR)-adjusted *P-*value < 0.05] (Fig. 2E). These findings suggest that *P. mirabilis* possesses species-specific genetic determinants for signal transduction and secondary metabolism that may underlie its distinct biological properties compared to other *Proteus* spp.

Among the 24 species-specific core gene families (Table 1)*,* 8 were associated with fimbrial synthesis, consistent with previous reports of expanded and conserved fimbrial loci in *P. mirabilis* (22). This extensive fimbrial repertoire likely facilitates epithelial and catheter surface adhesion during UTIs. Four gene loci (*PMI0289-PMI0290*, *PMI2644-PMI2645*, *PMI2913-PMI2914*, *PMI3598-PMI3599*) encoded functionally diverse proteins: β-phosphoglucomutase/glycosyl hydrolase, membrane protein, filamentous hemagglutinin, and AcrAB multidrug efflux pump. Notably, hemagglutinin, as a key virulence factor, contributes to pathogenic infection by mediating adherence to host epithelia and suppressing the initial inflammatory response to infection, thereby promoting bacterial persistence (23). The *P. mirabilis* AcrAB efflux system appears to be associated with fluoroquinolone resistance and the intrinsic tigecycline resistance (24, 25). Two genes (*PMI1324* and *PMI3020*) encoded putative lipoproteins; one gene (*PMI0306*) encoded a putrescine-ornithine antiporter PotE; one gene (*PMI1728*) encoded a putative tryptophanyl-tRNA synthetase; the remaining four genes encoded unknown proteins. Among them, PotE is a transporter of putrescine, which has been shown to be a key extracellular signal required for swarming in *P. mirabilis* (26). Overall, this repertoire is conserved and species-specific, resulting in unique capabilities in *P. mirabilis*, particularly in terms of host adaptation, pathogenicity, and drug resistance. The identified species-specific core gene families could be considered candidate molecular targets for specific detection of *P. mirabilis* strains.

**TABLE 1:** *P. mirabilis* species-specific core gene families identified in the *Proteus* pan-genome

Locus tag	Gene	Production	Strand	Start	End
PMI0239	−^*a*^	Conserved hypothetical protein	+	285544	285990
PMI0289	*pgmB*	Beta-phosphoglucomutase	−	344579	345223
PMI0290	−	Glycosyl hydrolase	−	345216	347933
PMI0306	*potE*	Putrescine-ornithine antiporter	−	361271	362599
PMI0978	−	Conserved hypothetical protein	−	1049452	1049994
PMI1324	−	Putative lipoprotein	−	1400402	1401148
ORF1	−	Hypothetical protein	−	1439825	1439917
PMI1728	−	Putative tryptophanyl-tRNA synthetase	−	1842876	1843895
PMI1812	−	Putative fimbrial adapter	+	1944497	1945192
ORF2	−	Hypothetical protein	+	2337409	2337501
PMI2216	−	Putative fimbrial adhesin	−	2405304	2406323
PMI2218	−	Fimbrial subunit	−	2406933	2407499
PMI2219	−	Putative fimbrial protein	−	2407515	2408018
PMI2644	−	Putative membrane protein	−	2886711	2887889
PMI2645	−	Putative membrane protein	−	2887886	2888482
PMI2913	−	Putative holo-[acyl]-carrier protein	−	3193893	3194261
PMI2914	−	Putative hemagglutinin	−	3194336	3196852
PMI3020	−	Probable lipoprotein	−	3315888	3316691
PMI3086	−	Fimbrial chaperone	−	3389033	3389707
PMI3087	−	Outer membrane fimbrial usher protein	−	3389729	3392130
PMI3089	−	Putative fimbrial subunit	−	3392155	3392793
PMI3091	−	Putative fimbrial subunit	−	3394154	3394810
PMI3598	−	Multidrug efflux pump	+	3933031	3934170
PMI3599	−	Multidrug efflux pump	+	3934196	3937330

^*a*^ − indicates that the gene has not been assigned a standard name in the annotated genome.

### Identification and validation of novel species-specific molecular targets for *P. mirabilis*

The reference sequences (*P. mirabilis* HI4320 NC_010554.1) of 24 species-specific core gene families were subjected to BLASTn searches against our original, unfiltered set of 679 genomes (Fig. S1) and the NCBI core nucleotide (core nt) database (accessed on 27 August 2024) with the exclusion of *P. mirabilis* (taxid: 584) to confirm specificity. Following the exclusion of genes with homologs in other bacterial species, primers and probes were designed for the remaining candidates. *In silico* specificity validation was performed using Primer-BLAST searches of the NCBI core nt database with the exclusion of *P. mirabilis* (taxid: 584) to identify non-specific primers. Finally, the *in silico* specificity of two species-specific core gene families (reference sequences: *PMI3020* and *PMI3598*) for *P. mirabilis* was ascertained and the corresponding primers (Table 2) were synthesized. This *in silico* validation underscores a key advantage of our genome-mining approach over methods that rely on single-gene targets (e.g., *ureR*), which may have homologs in related species such as *Providencia stuartii*, potentially compromising specificity (11).

**TABLE 2:** Primers used in conventional PCR

Gene		PMI3020	PMI3598
Primer	F^*a*^	5′-TGGTGCTACTGTCGCTATTT-3′	5′-ACGGCATCTTATCGTGTCTC-3′
	R^*a*^	5′-TATCTTTGTCTATGGCTCTGG-3′	5′-CTGGCTTTGTCCACTACTCACT-3′
Product size		332 bp	384 bp

^*a*^ F, forward; R, reverse.

**Fig 3:**
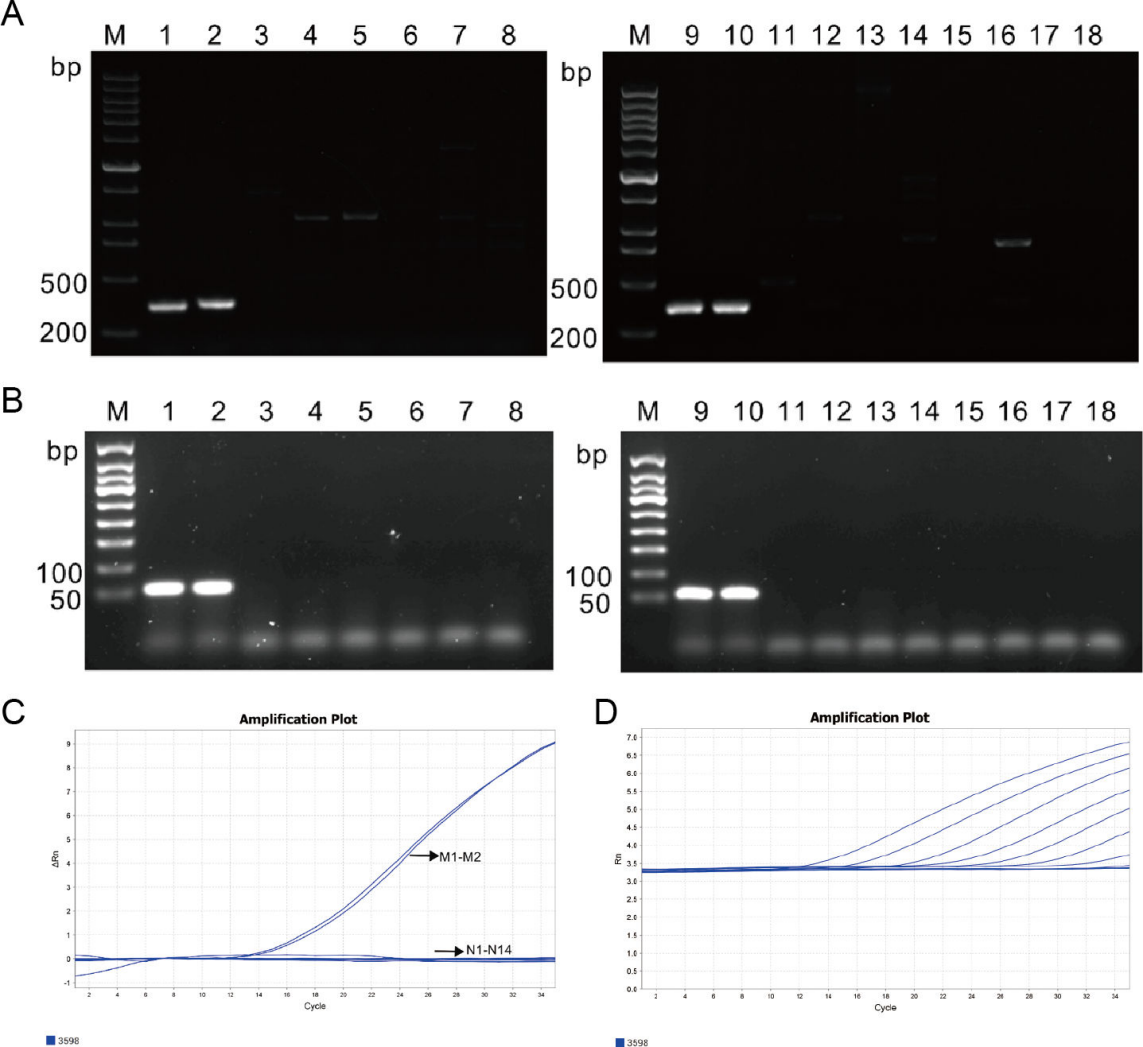
Specificity and sensitivity assessment of *P. mirabilis* in conventional PCR assay and TaqMan probe-based real-time PCR assay. Conventional PCR amplifications using *P. mirabilis* isolates, other *Proteus* spp. isolates, and non-*Proteus* isolates as templates and *PMI3020* (**A**) and *PMI3598* (**B**) as primers. A and B include DNA marker (lane M), *P. mirabilis* HI4320 (lanes 1 and 9), *P. mirabilis* DP2019 (lanes 2 and 10), *P. hauseri* ATCC 13315 (lane 3), *P. myxofaciens* ATCC 19692 (lane 4), *P. vulgaris* ACCC 11002 (lane 5), *Morganella morganii* ACCC 60117 (lane 6), *Providencia rettgeri* ATCC 29944 (lane 7), *Providencia stuartii* CICC 21520 (lane 8), *Escherichia coli* ATCC 25922 (lane 11), *Klebsiella pneumoniae* ATCC 700603 (lane 12), *Pseudomonas aeruginosa* ATCC 27853 (lane 13), *Acinetobacter baumannii* ATCC 19606 (lane 14), *Staphylococcus aureus* ATCC 29213 (lane 15), *Enterococcus faecalis* ATCC 29212 (lane 16), *Streptococcus agalactiae* group B ATCC 12386 (lane 17), and *Candida albicans* ATCC 14053 (lane 18). (**C**) Amplification plot of two *P. mirabilis* isolates and other 12 non*-P*. *mirabilis* isolates used in the experiment to assess the specificity of a TaqMan probe-based real-time PCR assay. Two *P. mirabilis* isolates showed a typical “S” amplification curve in the FAM channel, while the other isolates did not show a typical amplification curve. All data points are from an average of three technical replicates. (**D**) Amplification plot of different concentrations of *P. mirabilis* HI4320 used in the experiment to assess the sensitivity of a TaqMan probe-based real-time PCR assay. The FAM channel was used to detect *P. mirabilis*, and the concentration of the “S” amplification curve from left to right was in the range of 3.43 × 10^9^–3.43 × 10^2^ CFU/mL. When the concentrations of *P. mirabilis* HI4320 were 3.43 × 10^0^ and 3.43 × 10^1^ CFU/mL, no amplification curve was obtained. All data points are from an average of three technical replicates.

Two species-specific primers were applied to crude DNA from *P. mirabilis* isolates via conventional diagnostic PCR assay using DNA from two *P. mirabilis* isolates (HI4320 and DP2019) as positive controls and the other 14 non*-P*. *mirabilis* isolates as negative controls (Table S2), respectively. As expected, the *PMI3020* primers generated proper detectable amplicons (a 332 bp DNA product) in the two *P. mirabilis* isolates, but no specific products were obtained from the other 14 non*-P*. *mirabilis* isolates (including a *P. hauseri* isolate, a *P. myxofaciens* isolate, a *P. vulgaris* isolate) (Fig. 3A and B). Similarly, using the *PMI3598* primers, an expected 384 bp DNA product was amplified from *P. mirabilis* isolates but not from non*-P*. *mirabilis* isolates (Fig. 3C and D). Both species-specific primer sets are suitable for conventional PCR-based detection of *P. mirabilis*. The results with no cross-reactivity highlight the diagnostic reliability of our novel targets, offering a level of specificity that is challenging to achieve with phenotypic methods like MALDI-TOF or culture for closely related species.

Given its superior specificity (absence of nonspecific PCR products in 14 non*-P*. *mirabilis* isolates), the target gene *PMI3598* was further employed for developing a TaqMan probe-based real-time PCR assay (Table 3). This assay was designed to leverage the superior sensitivity and quantitative capability of real-time PCR over conventional methods (12, 13). The two *P. mirabilis* isolates exhibited a typical “S” amplification curve in the FAM channel, while the other 14 non*-P*. *mirabilis* isolates showed no typical amplification curve, indicating high specificity for *P. mirabilis* detection (Fig. 3E). Sensitivity was assessed using 10-fold serial dilutions of *P. mirabilis* HI4320 (3.43 × 10⁰–10⁹ CFU/mL) (Table 4). The reproducibility of the developed TaqMan real-time PCR assay was evaluated by intra-assay and inter-assay variability experiments. The standard deviation (SD) and coefficient of variation (CV) of Cycle threshold (Ct) values were ranged from 0.04 to 0.19 and from 0.15% to 0.55% in intra-assay, whereas they were varied from 0.03 to 0.25 and from 0.13% to 1.29% in inter-assay, respectively (Table 5). The findings suggest that the established real-time PCR assay exhibits satisfactory intra- and inter-reproducibility.

**TABLE 3:** Primers and probe used in TaqMan-based real-time PCR

Gene		PMI3598
Primer	F^*a*^	5′-GCCCTTTTCCCTTATGACACAAC-3′
	R^*a*^	5′-ATCGTTCTTACCACAGAGCTAATGG-3′
Probe		5'−6FAM-CCTTCTGTAAAATCG-MGB-3′

^*a*^ F, forward; R, reverse.

**TABLE 4:** Sensitivity of TaqMan-based real-time PCR assay

Concentration of *P. mirabilis* HI4320 (CFU/mL)	Cycle threshold (Ct)
(3.43 ± 0.99) × 10^9^	12.21 ± 1.06
(3.43 ± 0.99) × 10^8^	14.91 ± 0.43
(3.43 ± 0.99) × 10^7^	18.85 ± 0.33
(3.43 ± 0.99) × 10^6^	21.82 ± 0.24
(3.43 ± 0.99) × 10^5^	24.86 ± 0.43
(3.43 ± 0.99) × 10^4^	28.52 ± 0.74
(3.43 ± 0.99) × 10^3^	32.03 ± 1.17
(3.43 ± 0.99) × 10^2^	34.51 ± 0.34
(3.43 ± 0.99) × 10^1^	0.0
(3.43 ± 0.99) × 10^0^	0.0

**TABLE 5:** Intra- and inter-assay variability results of the developed TaqMan real-time PCR assay

Concentration of *P. mirabilis* HI4320 (CFU/mL)	Intra-assay		Inter-assay	
	Mean Ct ± SD	CV%	Mean Ct ± SD	CV%
4.18 × 10^3^	33.83 ± 0.19	0.55	33.26 ± 0.23	0.71
4.18 × 10^5^	26.01 ± 0.04	0.15	26.21 ± 0.03	0.13
4.18 × 10^7^	19.06 ± 0.04	0.23	19.71 ± 0.25	1.29

### AMR phenotypic prediction highlights MDR risk threat posed by *P. mirabilis*

**Fig 4:**
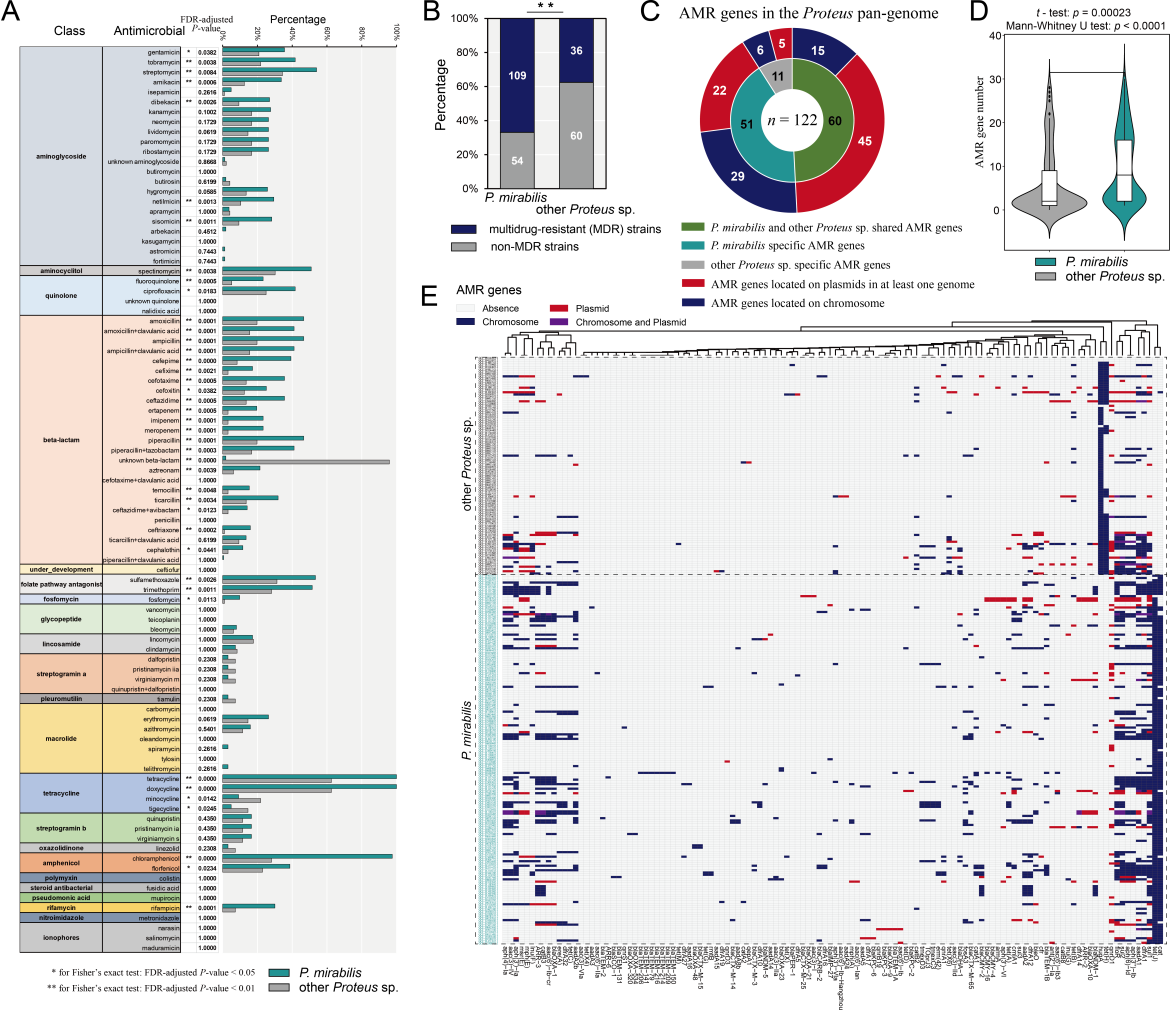
Phenotypic and genotypic prediction of antimicrobial resistance (AMR) for the genus *Proteus*. (**A**) Percentage of *P. mirabilis* genomes and other *Proteus* spp. genomes with an AMR phenotype using the WGS-based prediction tool ResFinder v4.3.1. Exact FDR-adjusted *P*-values (Benjamini-Hochberg method) from Fisher’s exact test are shown to the left of the bars for each antimicrobial. Asterisks indicate significance thresholds: * < 0.05, ** < 0.01. (**B**) Diagram illustrating the relationships between multidrug-resistant (MDR) strains in *P. mirabilis* and other *Proteus* spp. ** for Chi-squared test: *P*-value < 0.01. (**C**) Pie chart showing the distribution of AMR genes within the *Proteus* pan-genome. (**D**) Violin plot showing the relationships of AMR genes between *P. mirabilis* and other *Proteus* spp. (**E**) Heatmap displaying the distribution of AMR genes with the genome order aligned to the core genome tree (Fig. 1B). The colored blocks represent the presence in chromosomes or/and plasmid sequences, while the off-white blocks represent absence.

The growing antimicrobial resistance in *Proteus* spp. constitutes a critical public health challenge (27). Our WGS-based analysis using ResFinder v4.3.1 predicted resistance profiles for 91 antimicrobials across *Proteus* spp. (Table S3). While resistance was predicted for 71 antimicrobials, 20 showed no predicted resistance. Predicted resistance to the majority (65/71) of antimicrobials was found in both *P. mirabilis* and other *Proteus* spp. (Fig. 4A; Table S3). In contrast, predicted resistance to piperacillin-clavulanic acid, astromicin, fortimicin, arbekacin, spiramycin, and telithromycin was exclusively identified in a subset of *P. mirabilis* genomes and was absent from all other *Proteus* spp. genomes analyzed. All 163 *P. mirabilis* and most (94/96) other *Proteus* spp. strains were predicted to be resistant to at least one of the antimicrobial agents tested. Notably, *P. mirabilis* tended to have a higher rate of resistance [Fisher’s exact test: FDR-adjusted *P*-value < 0.01 or < 0.05] to almost half of the antimicrobials (41/91) than other *Proteus* spp. (Fig. 4A). The present findings are in accordance with those of a recent study, which reported *P. mirabilis* as a significant antimicrobial resistance threat, particularly with regard to carbapenems and quinolones (7). Multidrug resistance to three or more than three antimicrobial classes was observed more frequently in *P. mirabilis* (109/163) than in other *Proteus* spp. (36/96) [Chi-squared test: *P* < 0.0001] (Fig. 4B). *P. mirabilis* showed a high rate (>40%) of resistance to tetracycline (100%), doxycycline (100%), and chloramphenicol (97.5%), followed by streptomycin (54.0%), sulfamethoxazole (53.4%), trimethoprim (51.5%), spectinomycin (50.9%), amoxicillin (46.6%), ampicillin (46.6%), piperacillin (46.6%), tobramycin (41.7%), ciprofloxacin (41.7%), amoxicillin-clavulanic acid (41.7%), ampicillin-clavulanic acid (41.7%), and piperacillin-tazobactam (41.7%). Certainly, *P. mirabilis* has been increasingly reported to be resistant to extended-spectrum cephalosporins and co-resistant to aminoglycosides, fluoroquinolones, and sulfamethoxazole-trimethoprim (28, 29). For other *Proteus* spp., the most common resistance was to tetracycline (62.5%) and doxycycline (62.5%), with resistance to other antibiotics not exceeding 40%.

Initial use of broad-spectrum antimicrobials such as third- or fourth-generation cephalosporins and carbapenems is recommended for the treatment of UTIs (30). In this study, the resistance rate of *P. mirabilis* to cephalosporins ranged from 11% to 39%. However, no resistance was predicted for cefotaxime-clavulanic acid or ceftiofur in any of the *P. mirabilis* genomes analyzed. Furthermore, 23.3%, 23.3%, and 19.6% of *P. mirabilis* strains were predicted to be resistant to imipenem, meropenem, and ertapenem, respectively. Certainly, *P. mirabilis* isolates are increasingly reported to be resistant to extended-spectrum cephalosporins and carbapenems by acquiring resistance genes via mobile genetic elements (MGEs) (31, 32). The emergence of multidrug-resistant (MDR) *P. mirabilis* strains poses a significant threat to public health and highlights the urgent need for meticulous monitoring and surveillance of this pathogen. Furthermore, although ResFinder exhibited excellent concordance between genotypically predicted and phenotypically detected phenotypes, some inconsistencies remain (33, 34). Phenotypic validation through standardized antimicrobial susceptibility testing (AST) methods (e.g., broth microdilution or disk diffusion) remains the gold standard for confirming resistance and is crucial for translating genomic findings into clinical practice. Consequently, future experimental work to phenotypically validate these genotypic predictions, especially for important antimicrobials (such as carbapenems and cephalosporins), is essential.

### Emerging AMR genes enriched in the *P. mirabilis* genomes, with emphasis on carbapenemase encoding genes

To further investigate the potential genetic mechanisms of drug resistance, we investigated the distribution of AMR-related genetic determinants in the *Proteus* spp. genomes. A total of 122 AMR genes (containing allelic variants) were identified, corresponding to resistance to 15 antimicrobial classes, with primary resistance to beta-lactam, aminoglycoside, and tetracycline (Table S4). A total of 60 AMR genes were observed in both *P. mirabilis* and other *Proteus* spp. The majority of the remaining genes (*n* = 51) were found exclusively in *P. mirabilis*, and a limited number (*n* = 11) were found only in other *Proteus* spp. (Fig. 4C). On average, the *P. mirabilis* genomes contain 9.7 ± 8.2 AMR genes, exceeding the average of 6.0 ± 7.6 in the other *Proteus* spp. genomes [*t*-test: *P* < 0.01; Mann-Whitney *U* test: *P* < 0.01] (Fig. 4D). This significant difference in AMR gene abundance was consistently observed in our original, unfiltered set of 679 genomes (Fig. S2A). These AMR genes were scattered as non-core genetic elements throughout the *Proteus* spp. genomes, indicating their acquisition by HGT (Fig. 4E). Previous studies have shown that numerous AMR genes can be transferred between *P. mirabilis* and other Enterobacterales via various MGEs, including conjugative plasmids and integrative conjugative elements (ICEs) (35, 36). In this study, the presence of plasmid nucleotide sequences was detected in more than half (52.9%; 137/259) of the *Proteus* spp. genomes (Table S5). A total of 72 (59.0%; 72/122) AMR genes were found to be located on plasmid sequences in at least one genome (Fig. 4C). This finding indicates that plasmids play a pivotal role in the acquisition of AMR in *Proteus* spp., aligning with recent evidence that most resistance mechanisms in *P. mirabilis* are plasmid-mediated (7).

As shown in Fig. 4E, the most prevalent AMR genes in *P. mirabilis* were *tet*(J) (161/163) associated with doxycycline and tetracycline resistance and *cat* (150/163) associated with chloramphenicol resistance*.* In contrast, the *hugA* gene was present in most other *Proteus* spp. genomes (92/96). A total of 99 genomes, most of which (80/99) from *P. mirabilis*, harbored β-lactamase encoding genes. 37 genes/variants were grouped into 10 families, including *bla*_CARB_, *bla*_CMY_, *bla*_CTX-M_, *bla*_DHA_, *bla*_IMP_, *bla*_KPC_, *bla*_NDM_, *bla*_OXA_, *bla*_PER_, *bla*_SCO_, and *bla*_TEM_. Of these, *bla*_OXA-1_ (*n* = 37), *bla*_NDM-1_ (*n* = 26), *bla*_TEM-1B_ (*n* = 20), *bla*_CTX-M-65_ (*n* = 14), *bla*_CMY-2_ (*n* = 13), *bla*_OXA-10_ (*n* = 12), and *bla*_DHA-1_ (*n* = 11) were present in more than 10 genomes. The majority (28/37) of the β-lactamase encoding genes were present only in *P. mirabilis*; the remaining eight were found in both *P. mirabilis* and other *Proteus* spp., and one gene (*bla*_PER-1_) was found in two other *Proteus* spp. genomes. Twenty-five genes were predicted to confer resistance to carbapenems and/or cephalosporins, most of which were exclusively present in *P. mirabilis.* These genes, including *bla*_CMY-4_ (*n* = 2), *bla*_CMY-16_ (*n* = 3), *bla*_CTX-M-3_ (*n* = 5), *bla*_CTX-M-14_ (*n* = 4), *bla*_CTX-M-15_ (*n* = 2), *bla*_CTX-M-25_ (*n* = 1), *bla*_IMP-27_ (*n* = 3), *bla*_KPC-2_ (*n* = 2), *bla*_KPC-3_ (*n* = 1), *bla*_KPC-6_ (*n* = 1), *bla*_NDM-5_ (*n* = 2), *bla*_OXA-23_ (*n* = 4), *bla*_OXA-48_ (*n* = 2), *bla*_TEM-1A_ (*n* = 2), *bla*_TEM-2_ (*n* = 1), *bla*_TEM-131_ (*n* = 1), *bla*_TEM-141_ (*n* = 1), and *bla*_TEM-206_ (*n* = 1), were scattered throughout the *P. mirabilis* genomes. This is consistent with observations reported at the population genome level, which documented a 29.5% carbapenem resistance rate in *P. mirabilis* (7). In light of the fact that carbapenems and cephalosporins have been shown to be highly effective in the treatment of complicated urinary tract infections (cUTIs) caused by MDR strains, there has been an increasing prevalence of carbapenem- and/or cephalosporin-resistant *P. mirabilis* due to the acquisition of carbapenemase encoding genes (37–39). Consequently, developing innovative therapeutic approaches is critical for mitigating the rising threat of MDR *P. mirabilis* in clinical settings.

### Virulence-related genetic profile of the *Proteus* pan-genome

**Fig 5:**
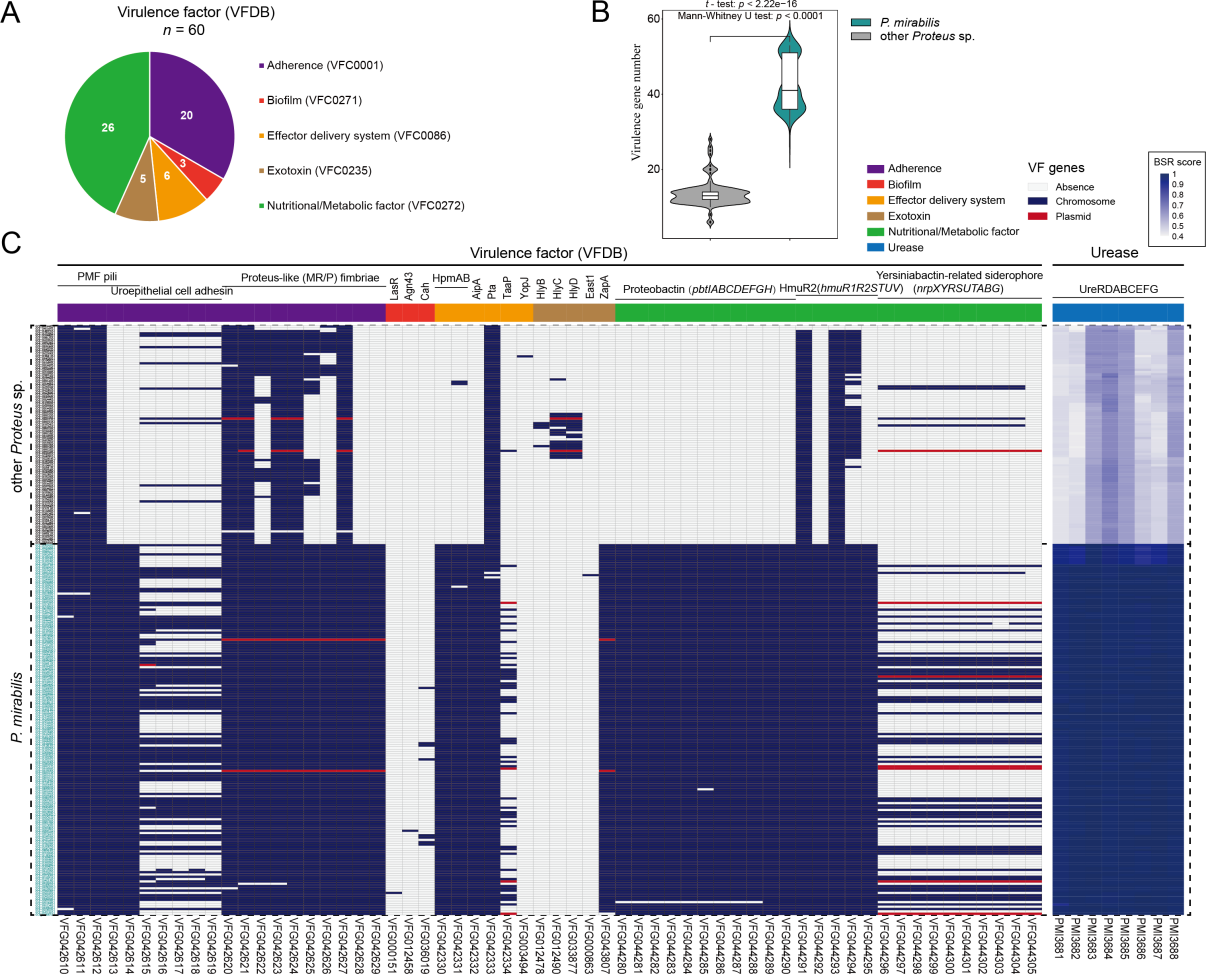
Distribution of virulence-related genes in the genus *Proteus*. (**A**) Distribution of virulence factor (VF) categories for the potential virulence-related genes. (**B**) Violin plot showing the relationships of virulence genes between *P. mirabilis* and other *Proteus* spp. (**C**) Heatmap displaying the distribution of virulence-related genes with the genome order aligned with the core genome tree (Fig. 1B). The colored blocks represent the presence in chromosomes or/and plasmid sequences, while the off-white blocks represent absence. The color coding of the blocks based on the blast score ratios (BSRs) that were calculated when the genomic data were screened against *PMI3681-PMI3688* of *P. mirabilis* HI4320.

*P. mirabilis*, known for its swarming motility and urease activity, is a frequent cause of catheter-associated urinary tract infections (CAUTIs) and has the potential to cause wound infections, rheumatoid arthritis, and meningitis in infants (4). To provide a comprehensive view of pathogenicity, we investigated the virulence-related genetic profile of *Proteus* spp. genomes. A total of 60 genes were found to match with known virulence genes in the VFDB database (Table S6), which were associated with “Adherence” (*n* = 20), “Biofilm” (*n* = 3), “Effector delivery system” (*n* = 6), “Exotoxin” (*n* = 5), and “Nutritional/Metabolic factor” (*n* = 26) (Fig. 5A). On average, the *P. mirabilis* genome contains 42.6 ± 6.7 virulence genes, exceeding the average of 13.7 ± 3.6 in the other *Proteus* spp. genomes [*t*-test: *P* < 0.01; Mann-Whitney *U* test: *P* < 0.01] (Fig. 5B). This significant enrichment of virulence-related genes in *P. mirabilis* was consistently observed in our original, unfiltered set of 679 genomes (Fig. S2B).

*P. mirabilis* fimbriae (PMF), uroepithelial cell adhesin (UCA), and mannose-resistant *Proteus*-like fimbriae (MR/P fimbriae) play an important role in biofilm formation and adherence to epithelial surfaces, which is essential for the pathogenicity of *Proteus* infections in both the urinary and gastrointestinal tracts (40). The complete biosynthetic loci of PMF (*pmf* locus) and MR/P fimbriae (*mrp* locus) are highly conserved in all *P. mirabilis* genomes (Fig. 5C). Notably, the nine genes comprising the *mrp* locus were the nine most upregulated genes during ascending murine UTI, as assessed by *in vivo* transcriptome analysis (Table S7) (41). The complete UCA biosynthetic locus (*uca* locus) was scattered among 87/163 *P. mirabilis* genomes and 9/96 other *Proteus* spp. genomes. Regarding “Nutritional/Metabolic factor,” *pbtIABCDEFGH*, *nrpXYRSUTABG*, and *hmuR1R2STUV* are involved in the biosynthesis of proteobactin and yersiniabactin related siderophore and hemin uptake systems, respectively. These iron acquisition and storage systems are reported to be strongly required during polymicrobial CAUTIs, contributing to the iron acquisition capabilities of *P. mirabilis* and promoting competition with other species (42, 43). Apart from the absence of *pbtIABCDEFGH* in a single strain, the complete *pbt* and *hmuR* loci are highly conserved in all *P. mirabilis* genomes. The *nrp* locus was scattered in 59/163 *P. mirabilis* genomes and 5/96 other *Proteus* spp. genomes, integrated in their chromosomes or carried by plasmid sequences (Fig. 5C). The yersiniabactin biosynthetic locus, known as the High Pathogenicity Island (HPI), was transferred horizontally by conjugation plasmid, which is required for virulence of highly pathogenic *Yersinia* species in mice (44).

The exotoxin encoding gene *zapA* was detected in all *P. mirabilis* genomes and was absent from other *Proteus* spp. genomes. Its transcription was found to be increased in swarm (fold-change = 4.6) and consolidate (fold-change = 4.8) compared to broth (Table S7) (45). ZapA, as a metalloprotease, could degrade IgG, IgA1, and IgA2 and contribute to the evasion of the innate immune response during infection (46). In addition, several exotoxin-related genes potentially relevant to gastrointestinal pathogenesis were found (Fig. 5C). The *hpm* locus, encoding a two-partner secretion pathway, consists of *hpmA* (encoding a cell-associated hemolysin) and *hpmB* (encoding an activator and chaperone of HpmA) (47). Both genes were identified as infection-specific fitness factors in the spleen as determined by Tn-Seq (fold-change *hpmA* = 12.0 and *hpmB* = 8.8) (48), and *hpmA* was also found to have transcription increased in swarm (fold-change = 8.1) and consolidate (fold-change = 4.7) compared to broth (Table S7) (45). This locus was found in 162/163 *P. mirabilis* genomes. The putative *east1*, a homolog of the heat-stable enterotoxin encoding genes of enteroaggregative *Escherichia coli* (EAEC), was present in only one *P. mirabilis* genome. In contrast, three hemolysin encoding genes, *hlyB*, *hlyC*, and *hlyD*, were found in parts of other *Proteus* spp. genomes. These hemolysins may contribute to gastrointestinal pathogenesis through cytotoxicity to innate immune cells, induction of the NOD-like receptor protein 3 (NLRP3) inflammasome, and stimulation of interleukin-1β (IL-1β) and tumor necrosis factor α (TNF-α) release (49, 50). The presence of these virulence factors provides evidence for the role of *Proteus* spp. in the pathophysiology of gastrointestinal disease.

Rapid urea hydrolysis, as a prominent phenotype of *P. mirabilis* isolates, is thought to contribute to urinary stone formation through the action of the urease enzyme (51). In this study, we showed that the urease encoding locus (reference nucleotide sequences: *PMI3681–PMI3688*) was present in all *Proteus* spp. genomes. The homologs were highly conserved in *P. mirabilis* with high Blast Score Ratio (BSR) scores averaging 0.983 ± 0.017 and were divergent in other *Proteus* spp. with low BSR scores averaging 0.538 ± 0.082 (Fig. 5C). The amino acid identities of the homologs (UreRDABCEFG) within *P. mirabilis*, with an average of 99.7% ± 0.5%, were higher than those between *P. mirabilis* and other *Proteus* spp., with an average of 90.1% ± 6.9% (Table 6). The high conservation and prevalence of the urease encoding loci suggests the importance of urease activity for *P. mirabilis*. Functional validation of the urease locus in other *Proteus* spp. may contribute to a more comprehensive understanding of the function of urease in this genus.

**TABLE 6:** Mean and variance values of the amino acid identity of UreRDABCEFG within *P. mirabilis* and between *P. mirabilis* and other *Proteus* spp.

	Intraspecies *P. mirabilis*	*P. mirabilis* vs other *Proteus* spp.
UreR	100.0% ± 0.1%	97.9% ± 0.3%
UreD	99.8% ± 0.6%	82.3% ± 0.7%
UreA	99.6% ± 0.6%	79.1% ± 0.8%
UreB	99.9% ± 0.2%	97.0% ± 1.0%
UreC	99.9% ± 0.3%	95.1% ± 0.7%
UreE	99.9% ± 0.1%	95.8% ± 0.4%
UreF	99.7% ± 0.7%	88.5% ± 1.4%
UreG	99.3% ± 0.7%	85.3% ± 1.0%

### Conclusions

This study provides a comprehensive pan-genome analysis of the genus *Proteus*, focusing on key genetic properties of *P. mirabilis*, including species-specific gene repertoire, AMR genes, and virulence-related genes. The open pan-genome revealed extensive genetic diversity. We identified two species-specific core genes (*PMI3020* and *PMI3598*) as molecular targets and developed conventional and TaqMan-based real-time PCR assays, with a detection limit of 3.43 × 10² CFU/mL for *P. mirabilis.* This demonstrates the utility of pan-genome analysis for mining specific markers and developing accurate diagnostic tools.

Comparative genomic analysis showed significant differences in AMR and virulence gene repertoires between *P. mirabilis* and other *Proteus* spp., with *P. mirabilis* possessing a higher abundance of AMR genes. The sporadic occurrence of AMR genes suggests extensive horizontal gene transfer, highlighting concerns for cephalosporin and carbapenem resistance. Continuous surveillance and integration of genomic data with molecular diagnostics are essential to mitigate the public health threat posed by clinical MDR *P. mirabilis*. Future work should focus on large-scale genomic data analysis to understand resistance dynamics. It is important to note that the findings of this study, particularly those concerning AMR and virulence, are primarily based on *in silico* genomic predictions. While these computational analyses provide valuable insights, they underscore the need for future phenotypic validation through standardized antimicrobial susceptibility testing and *in vivo* models to fully confirm the functional implications of the identified genetic determinants.

## MATERIALS AND METHODS

### Genome collection and filtering

A comprehensive search for *Proteus* spp. genomes was conducted using the NCBI RefSeq database, accessed on 14 November 2022. The detailed information of each genome is listed in Table S1. The initial collection encompassed 679 genomes. The quality assessment of these genomes was conducted via CheckM v1.0.13 (52). The inclusion of fragmented and incomplete genomes leads to significant core gene loss, and contaminated sequences have major influence on the identification of accessory genes (53). Subsequently, we excluded genomes with an excessive number of contigs (>200), less than 99% completeness, or more than 10% contamination, retaining a set of 572 genomes. The highly similar strains could bias the results of the pan-genome diversity and the identification of strain-specific genes (54, 55). Pairwise ANI were calculated using fastANI v2.0 with the default parameters (56). We picked representative genomes and excluded other redundant genomes (ANI values > 99.8%) from the data set, resulting in a final collection of 259 high-quality representative *Proteus* spp. genomes (Table S1).

### Pan-genome analysis

Unified gene finding and re-annotation of all genomes were conducted using Prokka v1.14.5 software (57). Orthologous groups of protein families of pan-genome were delimited using OrthoFinder2 software with DIAMOND method (58, 59). The OrthoFinder output files (deposited in the Orthogroup_Sequences folder) were used to extract pan-genome families (the totality of all genes found across strains), core genome families (genes shared among all strains), accessory genome families (genes shared among more than one strain, but not in all), and strain-specific genes (genes found only in one strain). Curve fitting of the pan-genome was performed using a power-law regression based on Heap’s law (*n* = $кN^{\gamma }$) (60, 61), where *N* is the number of genomes, *κ* is a proportionality constant, and the growth exponent *γ* > 0 indicates an open pan-genome. A descriptive statistical analysis was generated using OriginPro 9 software with the Allometric1 model. The gene families of the pan-genome were functionally characterized by the COG functional category (62) using eggNOG-mapper 2.1.9 software (63). We tested whether the functional enrichment of *P. mirabilis* specific gene families is different from pan-gene families of the *Proteus* genus using Fisher’s exact test and corrected for FDR using the “p.adjust” function with the Benjamini-Hochberg (BH) method in R v4.2.3 (64).

### Phylogenetic analysis

Core genome phylogenetic analysis was performed using SNPs across single-copy core gene families extracted from the OrthoFinder output files. Nucleotide sequences of the single-copy core gene families (*n* = 1954) were extracted according to the protein accession numbers and then aligned using the MAFFT v7.508 software (65). The set of SNPs presented in single-copy core gene families was extracted and then integrated according to the arrangement of the genes on the *P. mirabilis* HI4320 genome (complete genome & reference genome). Considering that recombination could occur in the bacterial genome and misguide the phylogenetic analysis, we identified and removed the putative recombinational regions from the SNP set using ClonalFrameML v1.12 software (66). The maximum likelihood (ML) tree was constructed using MEGA 11 (67) with the general time reversible (GTR) model and 100 bootstrap replicates.

### Bacterial isolates for specificity assay and genomic DNA extraction

The bacterial strains used in this study are listed in Table S2, including two *P. mirabilis* isolates, one *Proteus hauseri* isolate, one *Proteus myxofaciens* isolate, one *Proteus vulgaris* isolate, and nine other bacterial isolates. *P. mirabilis* HI4320 (clinically isolated strain from a patient with urinary tract infection, Tet^r^) was kindly provided by Prof. Harry L. T. Mobley. *P. mirabilis* DP2019 was isolated from a urine sample of a 49-year-old female patient with urinary tract infection. All isolates were grown in Luria-Bertani (LB) media at 28°C until OD_600_ reached 0.8. Cultures were stored in 50% glycerol at −80°C until use.

Genomic DNA was extracted from the bacterial strains using the DNA extraction kit (GENFINE, China) according to the manufacturer’s specifications. DNA concentration was determined using a nanophotometer (Nanodrop, USA). Bacterial DNA was collected and stored at −20°C until used for PCR amplification.

### Primer and probe designing and PCR amplification

Homologs of species-specific core gene families were searched using the BLASTn searches of the NCBI core nucleotide (core nt) database with the exclusion of *P. mirabilis* (taxid: 584) (accessed 27 August 2024). Gene families with homologs in other bacterial species were then excluded. Primers and TaqMan probes (FAM labeled) for the remaining gene families were designed using Primer Premier 5.0 based on the *P. mirabilis* HI4320 chromosome sequence (NC_010554.1). Sequence features of each gene, such as regions of high or low GC-content and size, were examined to ensure equal amplification conditions. *In silico* verification was performed using Primer-BLAST searches of the NCBI core nt database with the exclusion of *P. mirabilis* (taxid: 584) to identify non-specific primers. Once specificity for *P. mirabilis* was ascertained *in silico*, the primers and TaqMan probes were synthesized by Shanghai (China) Sangon Biotech Co., Ltd.

Each PCR amplification was carried out in a total volume of 25 µL consisting of 2 µL template, 1 µL (10 µM) forward primer, 1 µL (10 µM) reverse primer, 12.5 µL 2× Taq Master Mix (Dye Plus), and 7.5 µL ddH_2_O. PCR amplification was performed on the BIO-CETER ReRure series. The amplification conditions were denaturation at 94°C for 3 min, followed by 30 cycles of denaturation at 94°C for 30 s, annealing at 58°C for 30 min and extension at 72°C for 1 min, followed by an extension at 72°C for 5 min. The PCR products were electrophoresed on a 1% agarose gel to determine the size of the DNA products. Cells from the bacterial strains listed in Table S2 were used as templates.

### TaqMan probe-based real-time PCR

Real-time PCR was carried out in Applied Biosystems QuantStudio 3 Real-Time PCR Systems. The amplification conditions were as follows: each 20 µL reaction mixture consisted of 2 µL template, 10 µL 2× Premix Ex Taq, 0.4 µL (10 µM) forward primer, 0.4 µL (10 µM) reverse primer, 1.2 µL (10 µM) probe, 0.2 µL 50× Rox Reference Dye II, and 5.8 µL diethyl pyrocarbonate (DEPC) treated ddH_2_O. After denaturation at 95°C for 30 s, the reaction mixture was run through 35 cycles of denaturation at 95°C for 5 s, annealing and extension at 62°C for 34 s. Fluorescence from the FAM channel was collected during the extension step of each cycle.

The 10-fold serial dilutions of genomic DNA from an isolate of *P. mirabilis* HI4320 were used to assess the analytical sensitivity of the TaqMan real-time PCR assay. A colony of *P. mirabilis* HI4320 on LB agar was expanded by growing cultures overnight at 37°C. Cells were harvested by centrifugation at 12,000 rpm for 2 min. Cell pellets were washed twice and resuspended in PBS. Serial dilutions of the cell suspension were used to obtain progressively different bacterial concentrations. *P. mirabilis* HI4320 populations were assessed by TaqMan real-time PCR. Three replicates were performed for each experiment. The reproducibility of the established real-time PCR assay was evaluated through intra-assay and inter-assay variability experiments. Genomic DNA extracted from *P. mirabilis* HI4320 at concentrations of 4.18 × 10³, 4.18 × 10⁵, and 4.18 × 10⁷ CFU/mL was utilized for this purpose. For intra-assay, each DNA concentration was analyzed in triplicate within the same PCR run. For inter-assay, triplicate measurements were performed for each DNA concentration across three independent experimental runs conducted on separate days. The Ct values were recorded, and the SD and CV were calculated in order to assess repeatability and reproducibility.

### Screening AMR genes, plasmid sequences, and virulence-related genes

AMR genes and phenotype prediction were carried out using ResFinder v4.3.1 (33), with a cutoff of 80% nucleotide identity and 80% nucleotide coverage. We tested whether the predicted AMR phenotypes of *P. mirabilis* are different from other *Proteus* spp. using Fisher’s exact test and corrected for FDR using the “p.adjust” function with the Benjamini-Hochberg (BH) method in R v4.2.3 (64). Plasmid nucleotide sequences were distinguished from assembled contigs or scaffolds via the GPU Docker image-based deeplasmid (68). Virulence genes were detected using Abricate v1.0.1 (https://github.com/tseemann/abricate), utilizing the Virulence Factors Database (VFDB) setB/full data set (69). Analysis of homologs related to the urease locus (*ureRDABCEFG*) was performed using the LS-BSR tool with default parameters (70). The formatted nucleotide sequences of *PMI3681* to *PMI3688* were used as a database for comparison, validating homologs with a BSR > 0.8.

## SUPPLEMENTAL MATERIAL

Fig. S1 aem.01898-25-s0001.jpg Genotypic profiles of 24 *P. mirabilis* species-specific core gene families across the *Proteus* genomes.

Fig. S2 aem.01898-25-s0002.tif Violin plot showing the relationships of AMR genes and virulence-related genes between *P. mirabilis* and other *Proteus* spp.

Supplemental legends aem.01898-25-s0003.docx Descriptions of Fig. S1 and S2.

Supplemental tables aem.01898-25-s0004.xlsx Tables S1 to S7.

ASM does not own the copyrights to Supplemental Material that may be linked to, or accessed through, an article. The authors have granted ASM a non-exclusive, world-wide license to publish the Supplemental Material files. Please contact the corresponding author directly for reuse.
