# Supplementary figures and images for: Insights into novel diagnostic assay development, antimicrobial resistance, and pathogenicity in Proteus mirabilis through pan-genome analysis
Source: Appl Environ Microbiol. 2026 Feb 24;92(3):e01898-25. doi: 10.1128/aem.01898-25 (PMC12997850; doi:10.1128/aem.01898-25)

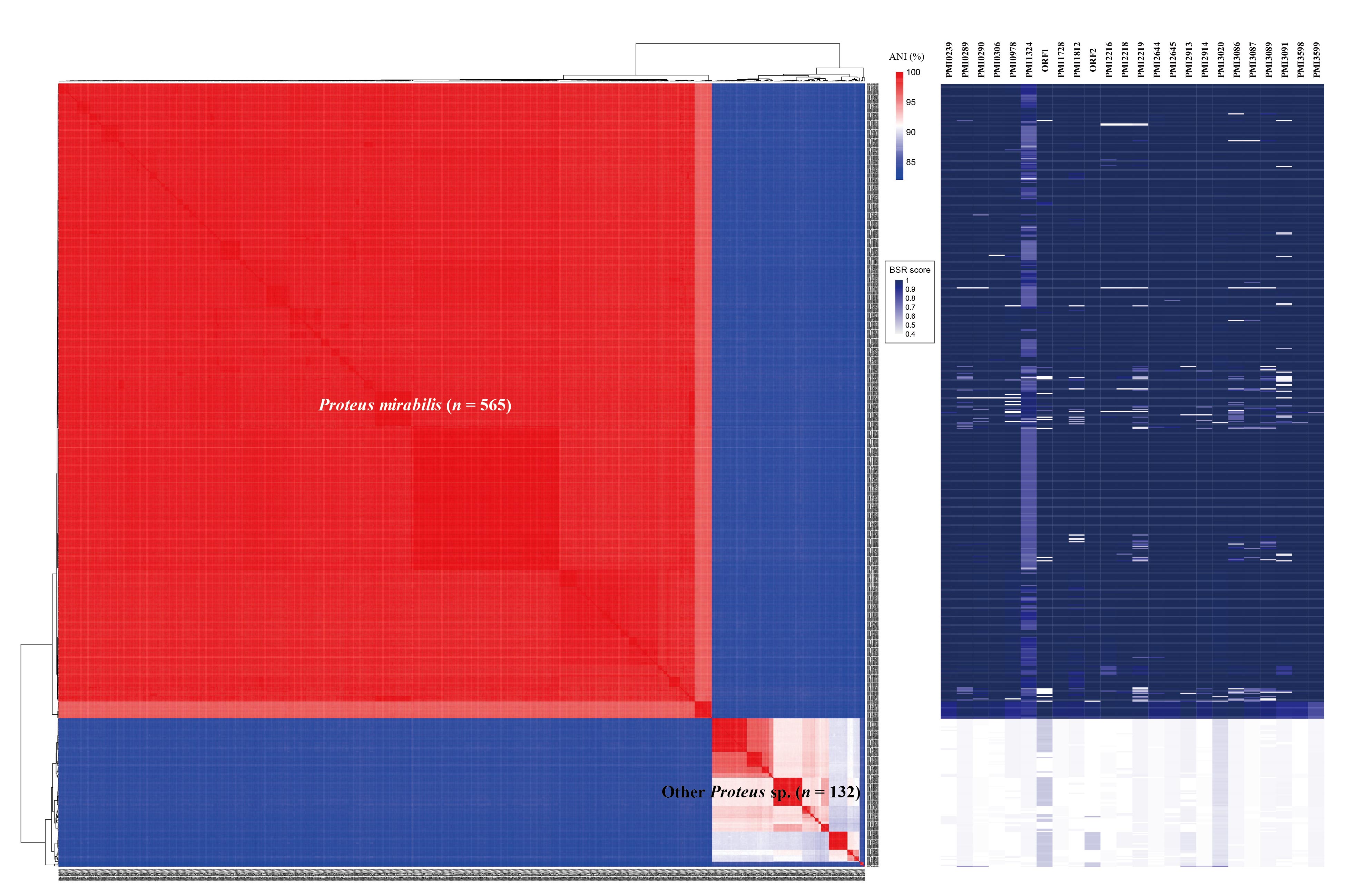

Supplement: Fig. S1 — Genotypic profiles of 24 P. mirabilis species-specific core gene families across the Proteus genomes. [file aem.01898-25-s0001.jpg]

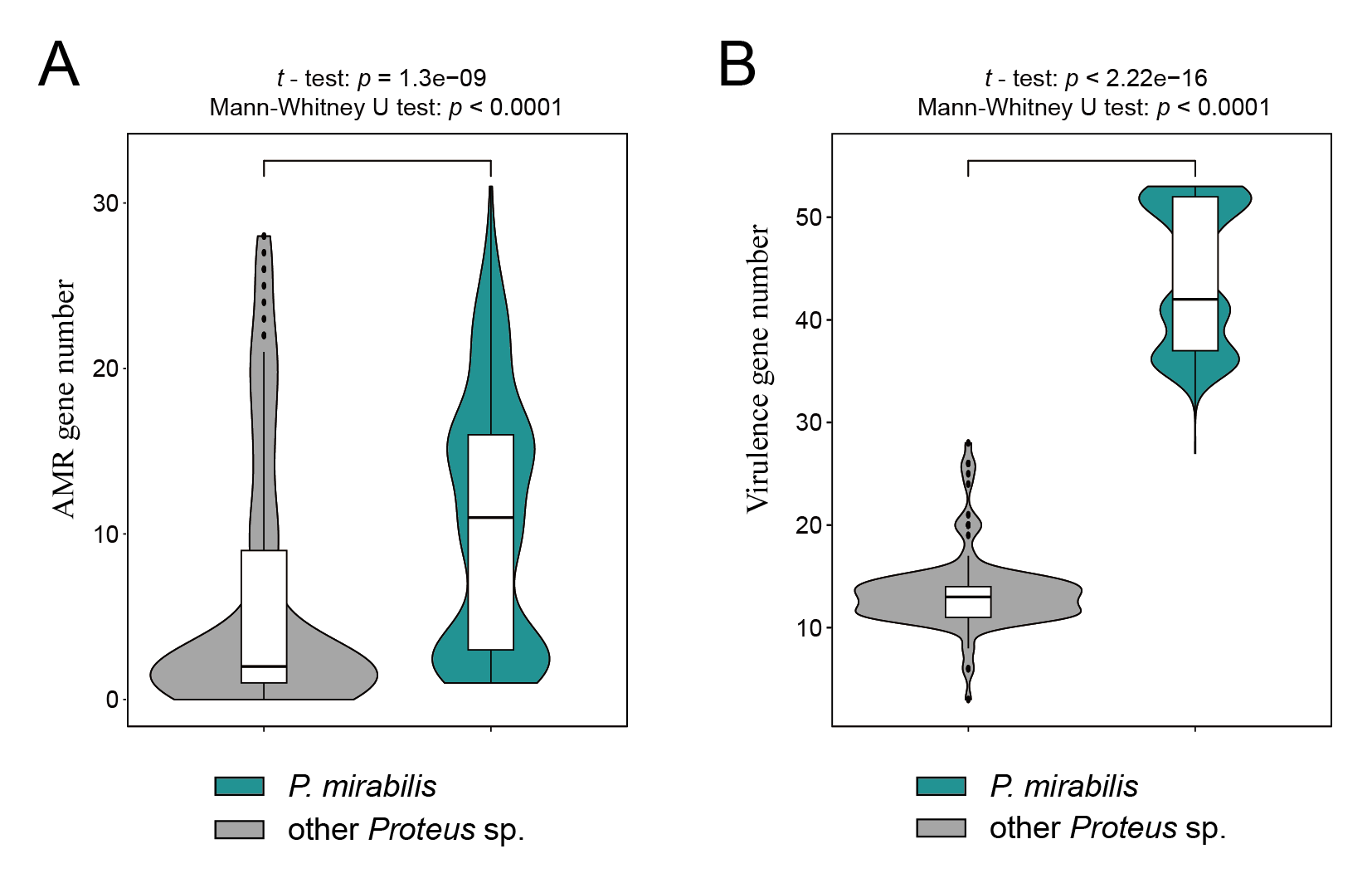

Supplement: Fig. S2 — Violin plot showing the relationships of AMR genes and virulence-related genes between P. mirabilis and other Proteus spp. [file aem.01898-25-s0002.tif]
