# Supplementary material for: Insights into novel diagnostic assay development, antimicrobial resistance, and pathogenicity in Proteus mirabilis through pan-genome analysis
Source: Appl Environ Microbiol. 2026 Feb 24;92(3):e01898-25. doi: 10.1128/aem.01898-25 (PMC12997850; doi:10.1128/aem.01898-25)
Supplement: Supplemental legends — Descriptions of Fig. S1 and S2. [file aem.01898-25-s0003.docx]

**Supplemental Material Figure Legends.**

**Figure S1.** **The genotypic profiles of 24 *P. mirabilis* species-specific core gene families across the *Proteus* (*n* = 697) genomes.** Pairwise average nucleotide identities (ANI) among the 697 *Proteus* spp. genomes, showing hierarchical clustering relationships. ANI values are represented by heatmaps, where similarity values are represented by the color key histograms on the right panels. The colored blocks next to the ANI heatmap indicate the distribution of *P. mirabilis* species-specific core gene families with the genome order aligned with the ANI heatmap. The color coding of the blocks is based on the blast score ratios (BSRs) that were calculated when the genomic data were screened against the species-specific core gene families within *P. mirabilis* HI4320.

**Figure S2.** Violin plot showing the relationships of AMR genes (A) and virulence-related genes (B) between *P. mirabilis* (*n* = 565) and other *Proteus* spp. (*n* = 132).
